# Emerging Concepts and Functions of Autophagy as a Regulator of Synaptic Components and Plasticity

**DOI:** 10.3390/cells8010034

**Published:** 2019-01-09

**Authors:** YongTian Liang

**Affiliations:** 1 Neurogenetik, Institut für Biologie, Freie Universität Berlin, 14195 Berlin, Germany; yongtian.liang@fu-berlin.de; Tel.: +49-3083856971; 2 NeuroCure, Cluster of Excellence, Charité Universitätsmedizin, 10117 Berlin, Germany

**Keywords:** autophagy, synapse, proteostasis, neurons, memory, aging, neurodegeneration, mitochondria, mitophagy, synaptic plasticity

## Abstract

Protein homeostasis (proteostasis) is crucial to the maintenance of neuronal integrity and function. As the contact sites between neurons, synapses rely heavily on precisely regulated protein-protein interactions to support synaptic transmission and plasticity processes. Autophagy is an effective degradative pathway that can digest cellular components and maintain cellular proteostasis. Perturbations of autophagy have been implicated in aging and neurodegeneration due to a failure to remove damaged proteins and defective organelles. Recent evidence has demonstrated that autophagosome formation is prominent at synaptic terminals and neuronal autophagy is regulated in a compartment-specific fashion. Moreover, synaptic components including synaptic proteins and vesicles, postsynaptic receptors and synaptic mitochondria are known to be degraded by autophagy, thereby contributing to the remodeling of synapses. Indeed, emerging studies indicate that modulation of autophagy may be required for different forms of synaptic plasticity and memory formation. In this review, I will discuss our current understanding of the important role of neuronal/synaptic autophagy in maintaining neuronal function by degrading synaptic components and try to propose a conceptual framework of how the degradation of synaptic components via autophagy might impact synaptic function and contribute to synaptic plasticity.

## 1. Introduction

Neurons are connected at specialized contact sites called synapses. These synaptic connections generate precise neural circuits and, thus, form the fundamental basis for various neuronal activities and brain functions including sensory perception, motor action, sleep, memory and emotion. Neural plasticity is mainly executed by structural and functional modification of synapses, whose plasticity requires tightly regulated protein synthesis and degradation in a spatial and temporal manner. Indeed, the proteome of the presynaptic and postsynaptic neurons functions in a highly dynamic and coordinated fashion to control neurotransmitter release. Quantitative proteomics combined with super-resolution imaging techniques have revealed that the synapse is extremely packed with proteins [1,2,3].

Neurons communicate by firing electrical impulses and must sustain this intense activity for a lifetime. Besides, neurons are post-mitotic cells with rare neurogenesis restricted to only certain neuronal populations and brain areas [4,5,6,7]. Furthermore, neurons are highly complex and polarized cells with extended axonal and dendritic processes, which adds another layer of complexity to the trafficking of proteins and organelles between the cell body and synaptic specializations. The extreme longevity of neurons throughout a lifetime of the individual [8,9] in the absence of pathogenic conditions undoubtedly poses a serious threat to preserve diverse molecular events including DNA repair and maintenance of protein, lipids and organelle homeostasis over time.

Naturally, one might ask how neurons and their synapses cope with these unfavorable circumstances while concurrently allowing for fine-tuned plasticity processes to preserve their functional stabilities and complexities. Clearly, neurons have evolved and adapted a sophisticated proteostasis network (PN) to accommodate their morphological complexities, high metabolic activities and longevity. Remodeling of the neuronal and synaptic proteome is accomplished by molecular chaperones, protein disaggregases and proteolytic pathways consisting of the ubiquitin-proteasome system (UPS) and autophagy-lysosome pathway (ALP). While it should be noted that there is an emerging paradigm of crosstalk between the UPS and ALP [10,11,12], ALP will be focused in this special issue in the context of autophagy as an emerging regulator of synaptic components and plasticity.

Autophagy, or ‘self-eating’ was termed by Nobel laureate Christian de Duve based on his observations of autophagic vacuole formation in the 1960s. Autophagy is an evolutionarily conserved bulk degradation process that sequesters cytoplasmic proteins, lipids, nucleic acids, polysaccharides and even organelles into double-membrane phagophores termed autophagosomes for subsequent lysosomal degradation. There are three distinct types of autophagy: chaperone-mediated autophagy (CMA), microautophagy, and macroautophagy (hereafter referred to as autophagy) [13,14,15].

Appreciation of the role of autophagy in both physiological and pathological brains continues to expand, as a plethora of evidence points out that appropriate regulation of autophagy is pivotal for neural integrity and central nervous system (CNS) development. Perturbations in the autophagy pathway are associated with neurodevelopmental and neurodegenerative diseases [16,17,18,19,20,21,22]. In recent years, evidence has been presented for the occurrence and requirement of autophagy at synapses [23,24,25,26], hinting at the potential regulation of synaptogenesis and refinement of synaptic connections via autophagy.

Compelling evidence has determined the spatial compartmentalized regulation of autophagy in neuronal sub-compartments [27,28]. Surprisingly, in addition to carrying degradative materials to the neuronal cell body for lysosomal degradation, autophagosomes also appear as conduits to relay neuronal signaling to the soma to promote neuronal functional complexities [29]. Furthermore, emerging studies have implicated the essential role of autophagy in neuronal homeostasis through the turnover of growing lists of disparately synaptic cargoes including synaptic vesicles, synaptic scaffold proteins and synaptic organelles [30,31,32,33]. That said, the regulation of autophagy might modulate synaptic structure, plasticity and function by changing the abundance of selective synaptic proteins in a spatiotemporal fashion, though its systemic role in the regulation of synaptic function has yet to be elucidated.

The purpose of this review is to recapitulate how autophagy is regulated at different sub-neuronal compartments to facilitate neuron-specific signaling, adaptations and functions, elucidate described and emerging synaptic components of autophagy, and highlight recent advances and emerging principles of how the regulation of autophagy might contribute to synaptic proteostasis and synaptic plasticity.

## 2. Compartment-Specific Regulation of Autophagy in Neurons: A Non-Canonical Role for Autophagosomes in Mediating Neuronal Signaling

To reiterate, neurons are unequivocally compartmentalized into the soma, dendrite, axon, pre- and post-synaptic regions. Accordingly, neuronal signaling is also highly compartmentalized, requiring sophisticated transport machineries to transmit and integrate information within and between sub-neuronal compartments [34]. Intuitively, to establish and maintain distinct neuronal domains, molecules and proteins must be generated and delivered in the right place at the right time. Active neuronal trafficking is crucial to ensure accurate distribution of numerous cellular cargoes [35]. Thus, the question arises whether and how the dynamics of autophagy are tailored in specific neuronal subdomains to facilitate compartment-specific demands and functions [36].

The dynamics, formation and maturation of autophagosomes in neurons have been worked out recently [27,28]. A seminal study using live-cell imaging of isolated dorsal root ganglion (DRG) neurons demonstrated that autophagosome biogenesis initiates distally at presynaptic terminals. Autophagosomes then undergo maturation during their retrograde transport to the cell soma, and form autolysosomes proximally [27]. Shedding more light on this issue, their follow-up work firmly established a compartment-specific mechanism of constitutive autophagy in hippocampal neurons, hinting at a possibly conserved machinery underlying autophagosome maturation along the axon in different neuronal subtypes [28]. However, it remains largely unknown how the spatial restriction of autophagosome biogenesis in distal axons is achieved. Together, this suggests autophagosome maturation state and motility may be differentially regulated in distinct sub-neuronal domains to meet their unique demands of cargo degradation.

**Figure 1:**
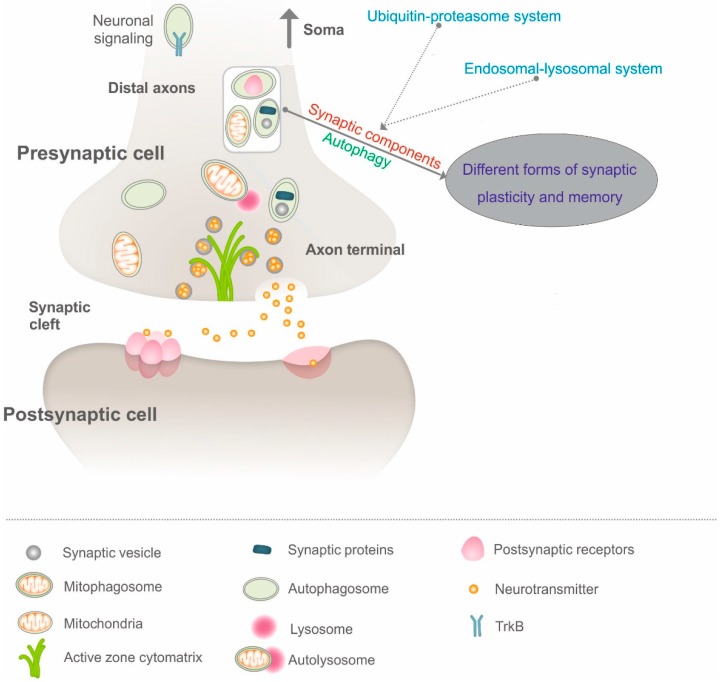
Emerging concepts and functions of how autophagy might regulate synaptic components and synaptic plasticity. Synaptic components (in red, outlined in the white rectangle within the presynapse) including synaptic proteins (PSD-95, PICK1 and SHANK3), synaptic vesicles, postsynaptic receptors (GABAA receptors and AMPA receptors following endocytic removal from the plasma membrane) and mitochondria are known to be degraded (straight line) by autophagy (in green), thereby potentially contributing to different forms synaptic plasticity (in purple, outlined in an oval-shaped frame) such as long-term potentiation (LDP), long-term depression (LTD) and memory formation. Ubiquitin-proteasome system (in blue) and endosomal-lysosomal system (in blue) also degrade (dotted lines) certain synaptic components and, thus contribute to synaptic plasticity and memory.

While the commonalities and divergence of synaptic autophagy and autophagy in other sub-neuronal compartments deserve to be investigated in detail and in various contexts, an exciting new twist on unanticipated ‘active signaling’ via autophagosomes has been discovered. While autophagosomes are retrogradely transported along the axon to the soma, they are found to simultaneously carry brain-derived neurotrophic factor (BDNF)-TrkB signaling (Figure 1), in effect, mediating neuronal complexities and preventing neurodegeneration [29]. Along these lines, the non-canonical role of autophagosomes as signaling organelles in stress and disease paradigms should be further investigated. Taken together, these ‘signaling’ autophagosomes represent a new paradigm distinct from their canonical role in degradation process. However, it could not be ruled out that there may exist multiple populations of autophagosomes ‘assigned’ with different tasks. To better discern this possibility, it may be necessary to continue efforts to characterize the membrane sources for autophagosome formation and track the differences of autophagosomes deriving from different neuronal sub-compartments.

Notably, biogenesis of autophagosomes and the initiation of their retrograde trafficking were also shown to be regulated by presynaptic activity [37,38]. It would be tempting to speculate that autophagosome biogenesis may also take place in other neuronal subdomains upon neuronal activity, though further investigations are needed. Recently, modulation of autophagy at the synapse and the rationale behind their interactions have attracted the most scrutiny [39,40]. The question arises as to what cargoes are degraded by autophagy at synaptic specializations.

## 3. Described and Emerging Synaptic Components of Autophagy

Synaptic cargo trafficking is vital for synapse formation, function and plasticity [41,42]. Previously recognized as a non-selective process, autophagy is now known to recognize and recruit specific cargoes via autophagy receptors for subsequent lysosomal degradation [43]. The degradation of selected cargoes at the synapse must follow a time- and space-dependent fashion to allow for specialized synaptic and neuronal function. Although what is engulfed in autophagosomes at synapses remains largely elusive, some well-defined and emerging cargoes at the synapse that are degraded by autophagy have been explored, including synaptic proteins and vesicles, postsynaptic receptors and organelles (including mitochondria) [44] (Figure 1).

Postsynaptic receptors, namely GABAA receptors and AMPA receptors, have been shown to be degraded by autophagy [45,46] (Figure 1). An implication of these findings suggests autophagy may serve as a candidate mechanism to regulate neuronal excitation-inhibition balance, which is important for synaptic plasticity and brain function.

Moreover, presynaptic autophagy has also been shown to modulate synaptic vesicle numbers and neurotransmission [47] (Figure 1), thereby potentially contributing to synaptic plasticity. Recently, Rab26, a protein on the surface of vesicles near synapses, has been shown to direct synaptic vesicles (SVs) to the autophagic program [48], although further work elucidating how this novel pathway is regulated remains to be scrutinized.

Notably, mitophagy (selective autophagy targeting mitochondria for degradation) (Figure 1) as a mitochondrial quality control mechanism at the synapse may be of vital importance because synaptic activities, such as axonal growth and branching, neurotransmission and long-distance cargo transport are energy-demanding and require proper calcium buffering capacity from mitochondria [49,50]. Burgeoning evidence has shown that degradation of mitochondria can occur in both the cell body and distal axons close to presynaptic specializations [27,51,52,53,54,55,56]. That said, although mature lysosomes with high degradative capacity reside primarily in the cell soma, degradation of cargoes in axonal domains can also occur locally. Indeed, axonal mitophagy can rapidly provide neuroprotection upon oxidative stress without arduous retrograde transport of damaged mitochondrial to the soma, ensuring mitochondrial homeostasis in a timely fashion. Conversely, defects in mitophagy may result in dysfunctional presynaptic mitochondria, which would impair synaptic homeostasis and, thus, culminate in neurodegeneration [57].

Recently, the contribution of autophagy to synaptic protein turnover has been deciphered. The Tavernarakis lab has shown postsynaptic density scaffolds PSD-95, PICK1 and SHANK3 to constitute autophagosomal cargoes (Figure 1), as these three synaptic proteins are present in purified autophagosome supernatant fraction in a western blot analysis [58]. Importantly, the exact mechanisms governing the selective targeting of synaptic proteins by autophagic degradation and whether they might be dynamically regulated by neuronal activity have yet to be deciphered. That the refinement of synaptic molecular composition and regulation of synaptic plasticity by autophagy steering the degradation of synaptic proteins appear possible (Figure 1).

Additionally, the mechanistic underpinnings of autophagy modulation by synaptic proteins have been shown. Several synaptic proteins have been found to modulate the rate of autophagosome biogenesis. The presynapse-enriched proteins EndophilinA (EndoA) and Synaptojanin-1 (Synj1) were found to interact with autophagy-related proteins to promote synaptic autophagy [59,60,61], independent of their known function in synaptic vesicle endocytosis [62,63]. Conversely, another presynaptic active zone (AZ) protein Bassoon sequesters key autophagy machinery Atg5 to inhibit presynaptic autophagy [64]. Furthermore, loss of Bassoon triggers presynaptic autophagy. The co-existence of positive and negative local regulators of presynaptic autophagy likely provides one candidate mechanism for dynamic switches during aging and neurodegenerative diseases. Interdisciplinary studies combining genetic, molecular and cellular biology approaches, and cutting-edge imaging tools in different model organisms might help uncover more principles as such [65,66,67,68,69]. Therefore, it is conceivable that other AZ proteins that interact with autophagy will be unveiled in future. Together, these AZ proteins might be part of a complex network that can timely modulate autophagy to eliminate dysfunctional components to allow for optimal synaptic performance, plasticity and maintenance.

## 4. Intersection of Autophagy and Synaptic Plasticity: Emerging Concepts and Relevance?

The capacity of synapses to undergo lasting morphological and biochemical changes and modify neural circuits in response to learning and neuromodulators is known as synaptic plasticity [70,71]. Precisely, these lasting changes in synaptic efficacy entail a broad spectrum of molecular modifications in presynaptic transmitter release, alterations in postsynaptic receptors, neuromodulator actions, the signal transduction pathways activated, gene activation and new protein synthesis [72]. Notably, increasingly sophisticated tools and methods have depicted that synapses are extremely plastic entities in both structure and function as a result of their high diversity in molecular makeup [73,74]. There are different forms of synaptic plasticity, including long-term potentiation and depression (LTP and LTD, respectively), homeostatic plasticity, metaplasticity, and spike-timing-dependent plasticity (STDP) [71,73]. Interestingly, functional diversity of synapses, such as the kinetics, strength or plasticity of synaptic transmission, often seems to mirror protein expression diversity [73]. Intrinsically, the bioenergetic and biosynthetic materials such as amino acids and other metabolic building blocks created from autophagic degradation can be used for new protein synthesis, which may be essential for a local control of synaptic plasticity in dendrites and axons apart from the cell body [75,76,77].

To date, it is widely accepted that both protein synthesis and protein turnover are required for synaptic plasticity [78,79,80,81,82,83]. Put differently, protein degradation is required to counterbalance protein synthesis, thereby allowing a tight control of local proteome at the neuron and the synapse to fine-tune synaptic connections during development and synaptic plasticity in adults. Research over the years has mainly focused on the proteolytic role of ubiquitin-proteasome system (UPS) and the endosomal-lysosomal system in neural plasticity and memory [84,85,86,87]. Remarkably, the UPS has been found to regulate presynaptic and postsynaptic proteins critical for neurotransmission and synaptic plasticity [87,88,89,90] (Figure 1), and the endosomal-lysosomal system could sort synaptic receptors for degradation in an activity-dependent manner [91] (Figure 1). In short, while degradation of synaptic proteins via the UPS has received considerable attention in the context of synaptic plasticity, the potential regulation of synaptic plasticity by autophagy is less explored and remains enigmatic.

Indeed, many different forms of long-term synaptic plasticity act together to shape the properties of neural circuits, but the most well-studied synaptic plasticity is NMDA receptor-dependent long-term potentiation (LTP) [92]. In contrast to long-term depression (LTD), long-term potentiation (LTP) represents a persistent increase in synaptic strength. Brain-derived neurotrophic factor (BDNF), a member of the neurotrophin family proteins, is an important regulator of long-term potentiation (LTP) in the hippocampus and in other brain regions [93,94]. In the aforementioned scenarios, autophagy has been found to modulate synaptic organization and plasticity by degrading postsynaptic receptors (GABAA receptors in a *Caenorhabditis elegans* study) and synaptic vesicles [23,45,46,47,48]. Moreover, in the Synj1-deficient zebrafish visual mutant *nrc^a14^*, loss of Synj1 (involved in synaptic vesicle cycling as mentioned above) leads to abnormal autophagic/endolysosomal activity in photoreceptor neurons [95,96]. Nikoletopoulou and colleagues further showed that suppression of autophagy is required for BDNF-induced synaptic plasticity. Interestingly, they discovered regulation of autophagy by fasting is varied across different brain regions, with induced autophagic activity in the hypothalamus and suppressed autophagic activity in the forebrain. This may lay a foundation for profound functional consequences in the brain. Importantly, this change in autophagic activity is paralleled by changes in BDNF levels. Furthermore, BDNF was found to suppress autophagy by transcriptionally downregulating key components (Atg12, LC3 and Gabarapl1) of the autophagic program. They then established that inhibiting autophagy is sufficient to rescue LTP defects imposed by loss of BDNF. Last, they showed that autophagy may modulate synapses by directly degrading synaptic proteins PSD-95, PICK1 and SHANK3, mutations in which have been implicated in autism spectrum disorders (ASD) [58]. Again, this elegant study supports the notion that autophagy may regulate synaptic plasticity by degrading synaptic components (Figure 1).

It is now widely believed that synaptic plasticity is the cellular mechanism for learning in mammals and other model organisms including the fruit fly *Drosophila melanogaster* [87]. Remarkably, supplementing a body-endogenous substance, called spermidine, was found to extend longevity across many species [97], and rescued age-related memory impairment (AMI) in *Drosophila* in an autophagy-dependent manner [98]. Mechanistically, spermidine blocks an age-associated ramp-up (synaptic strength reaching a ceiling level) in presynaptic proteins (Bruchpilot, Unc-13) and concomitant enhanced neurotransmitter release, which are causally related to the occurrence of AMI [99,100,101,102]. Despite this, how exactly autophagic regulations intersect with memory formation in *Drosophila* and in vertebrate models warrants further investigation. *Wdr45* (one of the orthologs of yeast *Atg18*) is essential for the formation of autophagosomes. Mutations in *Wdr45* cause ß-propeller protein-associated neurodegeneration (BPAN) characterized by cognitive impairments. Several studies showed that CNS-specific *Wdr45* knockout mice exhibit impaired memory, further supporting the role of autophagy in memory formation [103,104,105,106] (Figure 1).

In fact, several studies using rodent disease models have positively associated the upregulation of autophagy with the alleviation of synaptic plasticity deficits and cognitive impairments at the cellular and organismal level [107,108,109,110,111,112]. Despite the facts that these reports clearly suggest a link between autophagy and memory formation, an important caveat to bear in mind is that they were performed in the context of various diseases. Potentially relevant to these findings, Shehata and colleagues showed that in an auditory fear reconsolidation mice model, autophagy contributes to fear memory destabilization and induction of autophagy can be employed to augment the erasure of a reconsolidation-resistant auditory fear memory, providing a potential therapeutic opportunity for the treatment of anxiety disorders [113]. Taken together, it appears likely that autophagy may have an important role in certain forms of synaptic plasticity and memory formation (Figure 1), and this regulation may be conserved in many model organisms.

Although we are just scratching the surface, emerging links between autophagy and synaptic plasticity and how the deregulation of autophagy might cause synaptic defects and neurological disorders have been gradually revealed. Thus, future efforts targeting specific types and steps of the autophagic process may hold therapeutic potential for disease-modifying strategies [114].

## 5. Conclusions

As mentioned, all these concurrent formidable challenges faced by postmitotic neurons require various quality control pathways to maintain neuronal function. Autophagy, as a cellular quality control mechanism, plays an essential role in neuronal development, survival and homeostasis, as deregulation of autophagy has been implicated in neurodevelopmental disorders and neurodegeneration. Indeed, the surveillance and repair functions of autophagy are critical to safeguard neuronal synapses due to their high susceptibility to disturbance of proteostasis.

Convincing evidence has established compartment-specific regulation of autophagy in different neuronal subdomains, likely facilitating cargo degradation in a spatiotemporal manner. Apart from a conventional role in degradative pathways, a non-canonical function of autophagosomes in mediating neuronal signaling has been discovered. Furthermore, what contents are engulfed by autophagosomes at synapses have been deciphered, including synaptic proteins and vesicles, mitochondria and postsynaptic receptors. It is tempting to speculate that local degradation of synaptic components at the synapse and lysosomal degradation in the soma coexist to protect neuronal homeostasis under conditions of metabolic stress. Undoubtedly, degradation of synaptic components by autophagy plays a pivotal role in removing damaged synaptic proteins and organelles and, thus confers neuronal and synaptic proteostasis. Last, emerging evidence indicates that autophagy may be required for certain forms of synaptic plasticity and memory formation through the turnover of synaptic components in a spatiotemporal dependent fashion.

## 6. Future Perspective

The role of autophagy in synapse biology is a relatively new active research field moving forward. Lessons learnt from the interactions between UPS and synaptic plasticity can be employed to directly examine more details in the autophagy-synaptic plasticity axis in various contexts. Although many autophagic cargoes have been uncovered, the synapse-specific substrates for autophagy remain obscure. Thus, the imminent challenge is to completely characterize and define the synaptic components within autophagosomes at the presynaptic and postsynaptic sites and determine whether the degradation of these synaptic components is dynamically regulated by neural activity and/or synaptic plasticity in a spatiotemporal manner. Another key issue to be addressed would be whether autophagy can selectively degrade synaptic plasticity-related proteins (PRPs), whose turnover via autophagy controls synaptic plasticity. Likewise, a systemic elucidation of synaptic defects stemming from compromised autophagy should be appropriately addressed. Moreover, further investigation is warranted whether and how autophagy cooperates with other degradative pathways to fine-tune synaptic plasticity in neurons. Last, emerging concepts and relevance in autophagy and synaptic plasticity could indicate that harnessing synaptic autophagy might become crucial for protective strategies in the context of synaptopathies such as cognitive impairments, intellectual disability, autism spectrum disorders (ASDs), schizophrenia, and epilepsy. Consequently, delineating interactions between autophagy and synaptic plasticity is a prerequisite that may allow for direct modulation (upregulation or downregulation) of autophagy specifically at sub-synaptic compartments in future.
